# How to Perform a Post-Mortem Examination: The Experience of a Lifetime, and Its Practical Teaching

**Published:** 1907-10-12

**Authors:** Henry D. Littlejohn

**Affiliations:** late Professor of Forensic Medicine University of Edinburgh.


### Hospital Clinics.

### HOW TO PERFORM A POST-MORTEM EXAMINATION:

### The Experience of a Lifetime, and its Practical Teaching.

An address delivered at the Medical Graduates' College and Polyclinic, London,
By SIR HENRY D. LITTLEJOHN, M.D., LL.D.(Edin.), late Professor of Forensic Medicine University of Edinburgh.

(*Concluded from page 8.*)

The liver is most apt to rupture. There is a fad about teaching policemen and every kind of person methods of resuscitation. I am sorry to tell secrets out of the prison house, but in three cases in which a great muscular man had been working away at the body, the liver was found to be ruptured. Of course, I said nothing. In all cases the liver is apt to be very congested, and it has been laid down as a rule, which I wish to emphasise, that you should carefully see whether the liver has given way or not. Of course, examine the kidneys and the urine.

Now comes a very nice question, put by the Coroner: "Was the deceased under the influence of liquor at the time of the accident?" "So far as I can make out, sir, he was not." Again and again house surgeons of London hospitals have been reprimanded because they forgot to smell the man when he was brought in from the streets. It is most important to notice that. "Did you, sir, take any steps to ascertain the presence of alcohol?" "I am not fit to do that; I am not an expert. I do not think I could discover alcohol." "Have you preserved any portion of the body so that you can do it?" "No, I did not." It does not look well.

It was Dr. Percy, of the School of Mines, taught in Edinburgh, who first, by distillation, detected the presence of alcohol in the brain.

If I were asked what parts of the body would you recommend us to place at the disposal of the coroner, I would say first of all a portion of the brain. The coroner will ask, Why?, and you can then tell him about Percy. Secondly, collect the ventricular fluid. I would be pleased if, at an examination on a man who died shortly after a debauch, the examiner asked for a pipette to withdraw some of the ventricular fluid. Of course, we must have some blood, and a portion of the lungs, and we must have some urine. I have no hesitation in saying you will gain a great deal of *kudos*, even before our excellent coroners, if you attend to these points. You have produced the various substances; it is for the coroner to order an analysis.

### Be Cautious.

We early had the stomach ligatured and set aside; now cut the ligatures and see the contents. If it is a supposed case of poisoning what must you do? In cases of poisoning, practitioners are afraid of opening the stomach, and they quickly put it into some vessel. We cannot allow that, because the changes in the stomach, owing to putrefaction, come on quickly and are very marked. It is very important to notice those changes in colour, the results of putrefaction. You will never forget them. If you are sent into the country, and you find nothing to account for death, you may conclude from the colour that there is something peculiar about the stomach, and may request that a chemical analysis be made. The suspected party must be apprehended. The chemist takes eight or ten days to make his examination; he runs over the whole gamut of poisons and finds none. A wire comes from Government, "Discharge the prisoner." Do you mean to say that man in such a case on coming out of prison receives the same respect as before he went in? In his little street he hears a man say: "That fellow had a narrow squeak." If I had known I was going to be honoured by such an audience I would have had diagrams showing you cases of persons dying of phthisis, with the stomach showing putrefactive, so marked that one might say, "I think I am justified in having a chemical analysis made." You have an enormous power of endangering a man's character owing to want of acquaintance with such facts. You have not the time now to attend the pathological theatre, but we should insist upon those changes being taught; they are more important than all the usual pathological appearances.

### The Practitioner's Duty in the Case of Suspected Poisoning.

There is another question I must put to you: If insidious, chronic poisoning is suspected, what should the medical attendant do? Remember what happened to Dr. Pritchard, of Glasgow, who poisoned his wife. A doctor was called in to the case, and, strongly suspecting poisoning, wrote an anonymous letter, and the attention of the authorities was directed to it. In the meantime the lady died. If insidious chronic poisoning is suspected, what should the medical attendant do? There should be no writing of anonymous letters. He should first engage an intelligent nurse, because, in the course of my experience, an intelligent nurse can get hold of portions of food and vomit without directing attention. In all these cases the poisoner watches, and you must throw him off his guard. You must get a specimen of the urine. But you should never conduct chemical experiments. If you want to sleep soundly at nights, have nothing to do with chemistry. It is an enoromous subject. You are put into the witness box, and are asked difficult questions in organic chemistry. The Judge says, "I think it is hardly fair to the doctor. I do not see what bearing that has on the question." "My lord," says the barrister, "my poor client is being tried for his life, and I want to make out the competency or incompetency of this witness." There it is; you cannot get out of it. It is a most difficult position. "If you cannot answer, sir, think for a moment. You are here to give such evidence as will hang this poor fellow." I advise you to have nothing to do with chemical analysis. In Glasgow, for example, I saw two gentlemen, decent respectable men in general practice, and they were suddenly called upon to make a post-mortem examination on the body of a man who was undoubtedly poisoned. I doubt whether they ever made an examination before. They removed the stomach contents and placed them in a pickle-bottle found in the kitchen. All great persons were interested in that trial. Playfair told me he had been telegraphed for to point out to the Queen and Prince Albert at Kensington the constituents of the body, which were arranged in bottles. "But before you begin," said the Queen, "tell us about this trial which is coming on in Glasgow. Is she guilty?" All the eyes of Europe were on these two practitioners, and it was an awful exhibition. What they ought to have said—for here was the most commanding barrister in Edinburgh, a man of magnificent manner, who drew himself up to his full height, and put them through their facings—was, "Mr. Barrister, you mistake. I am not here as an expert. I am a general practitioner, who was called in to this case. I am here to tell what I saw. I cannot cross swords with you as to the niceties of expert evidence with regard to this special poison." But what did those poor fellows do? The body was exhumed, and they said it presented a markedly healthy appearance. But one said he thought there was a tinge of yellow. "A tinge of yellow? You call that jaundice?" "Yes," they said. "Is jaundice any evidence of arsenical poisoning?" "Yes." "Can you give me any authority for that?" "I think Taylor." "There is Taylor, show it to me." With the eyes of the entire Court on this man he could not find the arsenic reference. The Judge sitting on the Bench—never shall I forget his kindness, and if I meet him in another world I will thank him for what he did. He said, "It is the case of Marshall." The poor witness clutched at the reference to Marshall. Then says Inglis, "Remember you are giving evidence affecting the life of a lady. Is Marshall a case, or is it a book?" "It is a pamphlet." In all these cases be frank, gentlemen, and say, "I think you mistake; I am not an expert." But I notice that all general practitioners, when they come to be paid after a long trial, expect expert fees. I have felt for these men exceedingly. We secure an intelligent nurse, and we insist upon having a consultation. There was a consultation in the celebrated case of Mr. Wooler, who undoubtedly poisoned his wife, and the young gentleman assistant. Experts thought there might be poisoning, but they were not sure. A man Hazelwood was called in consultation, but could throw no light on it. It must have been like a comedy. They said, "What are we to do? We all suspect poisoning; we had better ring." They rang. "Is Mr. Wooler in?" "Yes, he is in." In he came, perfectly self-possessed. "Well, it is an awkward thing, but we suspect your wife is being poisoned." "Whom do you suspect?" "We suspect no one; not at all." He saw the men with whom he was dealing. They said, "We are going to give a little ammoniated tincture of iron by the bowel and the mouth, and we hope the best from it." Strange to say, the pulse became better. Ultimately, when the woman died, the whole body was found saturated with arsenic; and this arsenic, no doubt, came into the possession of Mr. Wooler because he had a basketful of poisons. He had an 8 oz. solution in a bottle, and that bottle could never afterwards be found. We had two of the greatest men giving evidence in that case. Taylor examined the body of Mrs. Wooler. Christison was also there. How did he get his hand in it? The assistant said, "We will try the reaction of copper and hydrochloric acid." They did, and got a deposit on the copper, but they could not tell what the deposit was due to. They put it in a tube, sealed it, and sent it to my old teacher, Christison. He could not tell whether it was poisoning, but he said, "If you will send me a small quantity of the lady's urine by return of post you will get my verdict." It was sent, and in five minutes he made out the poison. He wrote off at once, but the lady had died an hour before the letter arrived. Wooler was defended by the celebrated Serjeant Wilkins, who stood, it is said, as the original of Buzfuz in Pickwick. He was a very big man, and bellowed almost like a bull. He said his client Wooler was in the hands of enemies, and charged the doctors with having poisoned the lady. The assistant, in giving evidence, said that on a certain day he suspected poisoning. He was eager apparently to show that, as a young man fresh from his school, he had detected something before his senior, Jackson. What a turn of the screw when Wilkins said, "Now, sir, on that day when you suspected poisoning, what did you do? Did you tell the poor husband?" "No." "Did you tell the patient herself she was being poisoned?" "No." "Did you tell your master, Jackson, or did you do anything to save the life of this poor person?" How the witness escaped with his life I do not know. Wilkins was an awful man. You are very apt, in the witness box, to try to be a little better than you ought to be, to think you ought to be more cute than usual. Always remember Talleyrand's celebrated sentence, "Never be too zealous."

We have a consultation, and arrange for an examination of the urine. In that Wooler case a man who had nothing more to do with the case than you or I, formed the notion that the woman had been poisoned by injections of arsenic. Although there were trained nurses, Wooler gave these injections himself. And this man an outsider, thought, "If I could get possession of that syringe I would make my fortune, for I should know the avenue by which the arsenic reached the body of the person." If you pray incessantly to the gods even for a stick to break your heads you will ultimately get it. He got hold of the syringe. But little did he know what would happen. He washed the couplings and got a deposit on the copper rod. He met a friend, and told him he had discovered the avenue by which the arsenic was introduced. The friend said, "Are you sure your reagents were correct?" "Yes; I got them from the Apothecaries' Society of London." What did he ultimately discover? That the hydrochloric acid which he used contained arsenic. The friend said, "You introduced the arsenic into your experiment." Again, gentlemen, if you want to sleep soundly at night, have nothing to do with analysis.

### Avoid Anonymous Letters.

Next I should say, make a confidential statement to the Chief Constable: "I suspect that so and so is being slowly poisoned." There should be no anonymous letters. Say in the witness box, "I am not qualified to speak on those nice points, but anything I saw I shall be happy to mention." I remember very well in giving evidence about a case of effusion of blood into the brain, a volume of the "Lancet" was handed to me. The barrister said, "I suppose you are acquainted with that periodical, doctor?" "Yes, it is a very valuable periodical." "Would you pay a good deal of attention to any strong statement in that if it came from an accredited source?" "Undoubtedly." This is the statement; I have copied it out, and I want to give you the date, because it may be useful to you afterwards if you are speaking for the defence in a case of effusion of blood in the brain: January 26th, 1889, page 185: "Very little force is required to produce hæmorrhage in tissues whose nutrition has been long impaired by the action of alcoholic intoxicants." You agree with that, of course, and it is most important to remember that there is thus exaggeration in all effusions of blood in consequence. Again, an eminent professor, in the course of his lectures, says as follows: "In chronic alcoholism, fibroid degeneration of important organs is generally present." I have been asked if I agree with that. Yes, I do. And that tells in favour of the prisoner. The other quotation is from a clinical lecture given by an eminent professor, published in the "Medical Press and Circular," July 6th, 1892, page 12.

### Examine the Spine and the Cord.

Are any of our post-mortem examinations perfect? Not one. What is the important part of the body that is never examined? The spinal cord. And if the barrister knew what to say he would ask, "Well, did you make a perfect examination in this important case?" We rarely examine the spinal cord; there are two occasions in which we must examine it. That is to say, in poisoning with strychnine and in death from tetanus." Here you must do it. For what happened in the case of John Parsons Cook? A full examination was made of the body, and yet the body was exhumed three months afterwards to have the spinal cord examined? I should feel awfully annoyed if anybody which I had examined were to be exhumed afterwards. What was the state of the spinal cord when the body was exhumed? It must have been like cream by that time; what could you make out? What could you see? The theca and the dura mater of the cord resist putrefaction for, I should say, eight or nine months, and they wished to make sure that there were no atheromatous patches upon the theca of the cord. That might account for the tetanic spasms. One remarkable thing was made out in this exhumation. The hands of John Parsons Cook were clenched, the feet turned inwards, proving that the wonderful contracture induced by strychnine had remained three to four months.

### The Air Passages.

There is one part of the body which I have forgotten to speak about, and that is the air-passages. In every case the air-passages must be examined, because there may be a foreign body there, and it may be awkward if you omit this. But there is a far more important thing. If you are asked in the witness-box, "Is it not a most important part of the nervous system, this upper part of the cord at the cervical vertebræ?" "Yes." "It is so important that if touched with a knife the person is killed?" "Yes." "Did you examine that?" Lord Cockburn, one of our most eminent barristers cross-examined the medical men and got them to say that they never examined the upper portion of the cervical spine. "Therefore," said Cockburn, "there might have been injury or disease there?" "I don't think so." "But you cannot say, because you did not examine it." Now there is an easy way of assuring yourself of the integrity of the first two or three cervical vertebræ. You have removed the brain, and that enables you to pass your left forefinger down the foramen magnum and with your right hand on the vertebræ in front you can tell to a nicety the integrity of the spine.

### An Important Case to Remember.

I could go on for a couple of hours more, gentlemen; but allow me to tell you this. Who is one of the chief barristers who have come to the front in the last 20 years, and has recently given up political life? Sir Edward Clarke. How did he make his reputation? He said, "The first rung of the ladder I put my foot on was the well-known case, the so-called Penge case, not far from London." He put the first question, asking whether they had made proper search for the cause of death. "No." The second question was, "Did you examine the œsophagus?" "No." "There might then have been cancer, or stricture of the œsophagus." The third question was, "Did you examine the urine? There might have been diabetes." The urine had not been examined. Then he asked about the adrenals. There was marked discoloration of the skin, and yet they never examined the suprarenal capsules. A cardinal point in starvation is a thinning of the intestines. Would you believe it?—they were asked if they had seen it, and they answered "No." But there was some tubercle in the brain and membranes, and also in the lungs. The prisoner, though convicted, was at once pardoned and the sentence of death commuted. "And," said Clarke, "that was the case which put me on a fair road to fortune." It is very important to remember such a case.

